# Evidence Implicating the Ras Pathway in Multiple CD28 Costimulatory Functions in CD4^+^ T Cells

**DOI:** 10.1371/journal.pone.0024931

**Published:** 2011-09-19

**Authors:** Sujit V. Janardhan, Kesavannair Praveen, Reinhard Marks, Thomas F. Gajewski

**Affiliations:** 1 Department of Pathology, The University of Chicago, Chicago, Illinois, United States of America; 2 Department of Medicine, The University of Chicago, Chicago, Illinois, United States of America; University of Georgia, United States of America

## Abstract

CD28 costimulation is a critical event in the full activation of CD4^+^ T cells that augments cytokine gene transcription, promotes cytokine mRNA stability, prevents induction of anergy, increases cellular metabolism, and increases cell survival. However, despite extensive biochemical analysis of the signaling events downstream of CD28, molecular pathways sufficient to functionally replace the diverse aspects of CD28-mediated costimulation in normal T cells have not been identified. Ras/MAPK signaling is a critical pathway downstream of T cell receptor stimulation, but its role in CD28-mediated costimulation has been controversial. We observed that physiologic CD28 costimulation caused a relocalization of the RasGEF RasGRP to the T cell-APC interface by confocal microscopy. In whole cell biochemical analysis, CD28 cross-linking with either anti-CD28 antibody or B7.1-Ig augmented TCR-induced Ras activation. To determine whether Ras signaling was sufficient to functionally mimic CD28 costimulation, we utilized an adenoviral vector encoding constitutively active H-Ras (61L) to transduce normal, Coxsackie-Adenovirus Receptor (CAR) transgenic CD4^+^ T cells. Like costimulation via CD28, active Ras induced AKT, JNK and ERK phosphorylation. In addition, constitutive Ras signaling mimicked the ability of CD28 to costimulate IL-2 protein secretion, prevent anergy induction, increase glucose uptake, and promote cell survival. Importantly, we also found that active Ras mimicked the mechanism by which CD28 costimulates IL-2 production: by increasing IL-2 gene transcription, and promoting IL-2 mRNA stability. Finally, active Ras was able to induce IL-2 production when combined with ionomycin stimulation in a MEK-1-dependent fashion. Our results are consistent with a central role for Ras signaling in CD28-mediated costimulation.

## Introduction

Full activation of effector CD4^+^ T cells requires ligation of not only the T cell receptor (TCR) by peptide-MHC complexes expressed on antigen presenting cells but also engagement of costimulatory receptors. The most widely studied costimulatory receptor on T cells is CD28, ligation of which has been shown to have several critical and distinct effects on T cell activation in vitro and in vivo. In CD4^+^ T cells, CD28 costimulation augments TCR-induced transcription of IL-2 and other cytokine genes [1], promotes the stability of cytokine mRNAs [2], increases cellular metabolism [3], augments survival of stimulated cells [4], and prevents the induction of a hyporesponsive state known as anergy [5] that results when T cells are stimulated through the T cell receptor alone.

Despite the well established importance of CD28 costimulation and extensive biochemical analysis of CD28 signaling events, molecular pathways sufficient to replace the multiple CD28 functions have not been identified. The discovery of a PI3K binding site in the CD28 cytoplasmic tail has generated much interest in the role of the PI3K-AKT signaling pathway in CD28 costimulation [6]. Mutation of this binding site abrogated PI3K binding and CD28-mediated AKT activation, resulting in a failure to upregulate the anti-apoptotic protein Bcl-xL. However, this mutation had no effect on CD28-mediated costimulation of IL-2 production [7], [8]. Further insight into the role of AKT signaling in CD28 costimulation can be gleaned from studies of another costimulatory molecule expressed on T cells, ICOS (Inducible COStimulatory molecule on T cells). ICOS and CD28 share homology including a PI3K binding site. However, despite the fact that ICOS induces stronger activation of AKT than CD28, ICOS costimulation is unable to augment TCR-induced IL-2 production [9]. Together, these data argue that while the PI3K-AKT pathway may play a role in CD28-mediated costimulation of survival, it is neither sufficient nor required for certain other CD28-mediated functions. It should be noted that subsequent studies using over-expression of a constitutively active AKT mutant in CD28 deficient primary T cells have argued that signaling downstream of AKT is able to replace CD28 mediated costimulation of IL-2 production [10]. However, it is conceivable that the process of retroviral transduction used to introduce this mutant may have selected for cells that survived better and therefore produced greater IL-2. Importantly, there has been no single biochemical pathway identified to date that has been sufficient to mimic and/or functionally replace all CD28-mediated costimulatory functions.

The CD28 cytoplasmic tail has also been shown to interact with the adapter Grb2 [11], which predominantly binds the Ras guanine nucleotide exchange factor SOS [12]. This observation may indicate a role for Ras signaling in CD28 costimulation. Although initial studies reported that Ras activation was augmented by anti-CD28 antibody crosslinking, it subsequently became unclear whether ligation of CD28 with its natural ligands, B7.1 or B7.2, would have the same effect [13]. The possibility that Ras may contribute to CD28 costimulation resurfaced with additional studies of ICOS. One critical difference between ICOS and CD28 is that the YMNM motif in the CD28 cytoplasmic tail allows for binding of PI3K as well as Grb2 while the homologous region of ICOS (YMFM) is unable to bind Grb2. Interestingly, a single point mutation of the ICOS cytoplasmic tail (YMF→NM) that allowed for Grb2 binding was sufficient to allow this molecule to costimulate IL-2 production [14], suggesting a link between the ability of CD28 to activate Ras and its ability to costimulate IL-2 production. Importantly, maximal Ras activation can be achieved with strong TCR stimulation without providing the functional benefits of CD28 costimulation. This argues that if Ras signaling does play a role in CD28-mediated costimulation, it must be due to a qualitative, not just a quantitative change in Ras activation.

CD28 has also been reported to promote Protein Kinase C theta (PKC theta) recruitment to the central supramolecular activation cluster (cSMAC) at the T cell-antigen presenting cell (APC) interface [15]. This raises the possibility that other diacylglycerol (DAG)-mediated signaling events, such as recruitment of the Ras guanine nucleotide exchange factor (GEF) RasGRP, might also be promoted by CD28. The accumulating evidence that RasGRP is actually the major GEF for Ras activation in T cells [16], [17], [18] makes this hypothesis attractive.

Based on these considerations, we sought to determine whether activation of the Ras signaling pathway might be influenced by CD28 costimulation and if so, whether direct activation of Ras signaling might be sufficient to mimic or functionally replace CD28 costimulation. In order to avoid the potential pitfalls of selection conferred by retroviral transduction systems, we utilized adenoviral transduction of T cells from Coxsackie and adenovirus receptor (CAR) transgenic mice, thereby allowing acute assessment of functional consequences following introduction of mutant signaling molecules without a requirement for cell proliferation [19].

## Results

### CD28 costimulation qualitatively and quantitatively influences Ras activation

**Figure 1:**
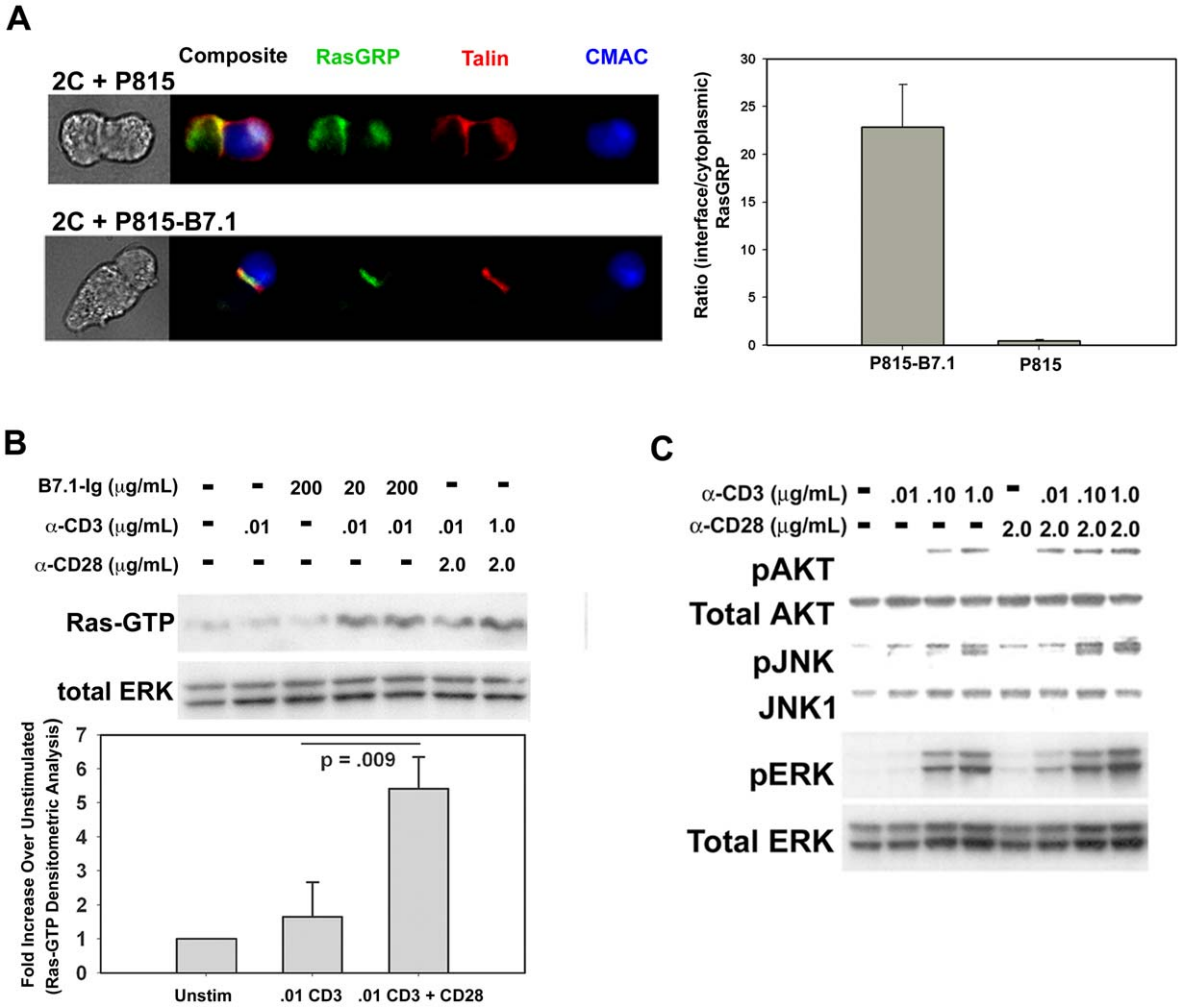
CD28-mediated costimulation relocalizes and augments Ras activation. **A.** Splenic CD8^+^ T cells from 2C TCR transgenic mice were primed in vitro as described [15] to generate effector cells. They were then incubated with CMAC-labeled P815 or P815-B7.1 tumor cells (expressing a ligand for the 2C TCR). Conjugates were stained for RasGRP and Talin and analyzed by confocal microscopy (left). Statistical analysis of T cell RasGRP localization was done by measuring the ratio of the average pixel intensity of RasGRP staining at the T cell-APC interface to the average pixel intensity of RasGRP staining in the T cell cytoplasm. Analysis of at least 100 conjugates per experiment demonstrates a significant relocalization of RasGRP to the T cell-APC interface when CD28 costimulation is added to P815 stimulation (p<0.05, right). See materials and methods for details of image analysis and criteria used to definine T-cell APC conjugate, T cell-APC interface and T cell cytoplasm. Error bars indicate the standard deviation of the mean pixel intensity ratios (interface/cytoplasm) under the two indicated conditions from the same experiment. B. CAR Tg Th1 T cells were stimulated with beads coated with anti-CD3 antibody alone, or with anti-CD3 plus either anti-CD28 or B7.1-Ig fusion protein for 30 minutes. Cellular lysates were analyzed for Ras activity as described in Materials and Methods (top). Ras activation was quantified using densitometric analysis of western blots to determine the fold-increase in RasGTP-generation above unstimulated cells (bottom). Error bars represent the standard deviation in mean Ras activation for a given stimulation condition between replicate experiments. The p-value indicated in the figure demonstrates a significant difference in Ras-GTP generation when CD28 costimulation was added to CD3 stimulation. **C.** CAR Tg Th1 cells were stimulated as in A with increasing doses of anti-CD3 (0.01–1 µg/mL) with or without anti CD28 (2 µg/mL) and analyzed for phosphorylated AKT, JNK, and ERK by western blotting.

Previous studies have shown the ability of CD28 to promote PKC theta localization to the cSMAC [20]. This observation raises the possibility that other DAG-dependent signaling molecules, such as the Ras GEF RasGRP, which has been shown to co-localize to areas of Ras activation [21], may also be influenced by CD28 costimulation. In order to begin to understand the role Ras signaling may play in CD28 costimulation, we first examined whether physiologic CD28 engagement could influence the nature of Ras activation by visualizing the cellular localization of RasGRP. Using 2C TCR transgenic T cells and P815 target cells transfected with the CD28 ligand, B7.1, we visualized RasGRP localization by confocal microscopy. In the absence of CD28-costimulation, RasGRP was primarily localized to cytoplasmic and/or intracellular compartments. In contrast, CD28-costimulation induced a relocalization of RasGRP to the T cell-APC interface (Figure 1A). Importantly, expression of B7.1 on P815 cells had no effect on the percent of T cells forming conjugates (data not shown). Additionally, the relocalization of RasGRP was not an artificial effect caused by photobleaching, as diffuse staining of RasGRP was seen in unconjugated T cells where photobleaching effects would still be present (data not shown). The observation that CD28 costimulation manipulates RasGRP localization within the T cell suggests that CD28 may in fact influence Ras activation by causing a relocalization of its key upstream activator.

Previous studies examining whether CD28 costimulation could result in a quantitative augmentation of Ras activation have given variable results [13]. In order to determine if Ras activation could be augmented by a natural CD28 ligand, we assessed whether CD28 ligation by either anti-CD28 monoclonal antibody (mAb) or by its natural ligand, B7.1 would augment Ras activation when co-ligated with the TCR complex. CD28 costimulation significantly augmented Ras activation induced by low-level TCR stimulation not only when CD28 was crosslinked by anti-CD28 mAb, but also by its natural ligand, B7.1 (Figure 1 B). No significant increase in Ras activation above unstimulated cells was seen in effector CD4^+^ T cells stimulated with B7.1-Ig or anti-CD28 alone, without TCR costimulation (p = 0.34, data not shown). As expected, augmented Ras activation was associated with augmented phosphorylation of AKT, JNK and ERK in concert with low doses of anti-CD3 mAb (Figure 1C). These results argue that CD28 costimulation can result in both a qualitative and a quantitative alteration in Ras-based signaling in T cells.

### Introduction of active Ras can mimic the ability of CD28 to augment IL-2 production, prevent anergy induction, promote cell survival, and increase glucose uptake

**Figure 2:**
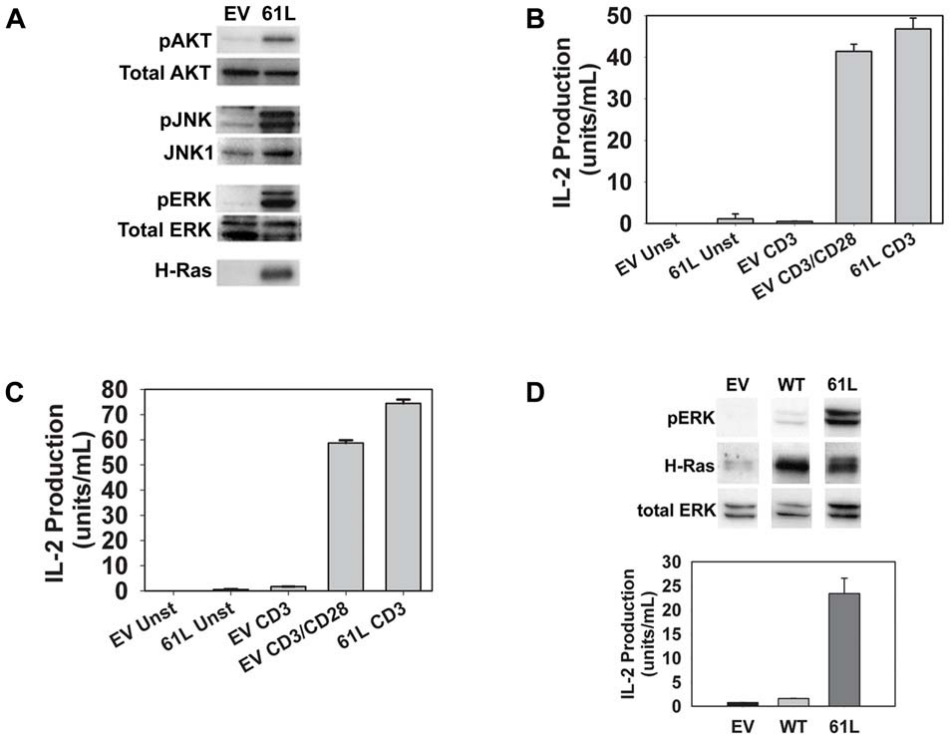
Transduction with active Ras can mimic CD28-mediated costimulation of TCR signaling and IL-2 production. **A.** CAR Tg Th1 T cells were transduced with adenoviruses encoding constitutively active H-Ras61L (61L) or an empty vector (EV) control as described in materials and methods. Cell lysates of unstimulated, transduced cells were analyzed for phosphorylated AKT, JNK, and ERK or H-Ras (the absence of H-Ras staining in EV transduced cells indicates the absence of H-Ras61L and/or significant amounts of endogenous H-Ras isoform in these cells; total Ras was abundantly detected in both EV and Ras transduced cells (data not shown)). **B.** CAR Tg Th1 T cells were transduced as in A and stimulated in triplicate overnight with beads coated with anti-CD3 antibody alone or with anti-CD28. Supernatants were collected and analyzed by ELISA for IL-2 production. **C.** Purified splenic CAR Tg CD4^+^ T cells were transduced, stimulated and analyzed as in B. **D.** CAR Tg Th1 T cells were transduced with adenoviruses encoding H-Ras61L (61L), wild type H-Ras (WT) or vector control. Transduced cells were either lysed and analyzed for protein expression (top) or stimulated in triplicate overnight with beads coated with anti-CD3 (bottom), and analyzed for IL-2 production by ELISA. Error bars in B, C, and D represent standard deviation of the mean values from triplicate samples from the same experiment. A significant increase in IL-2 production was seen when active Ras was added to CD3 stimulation in B, C, and D (p<0.05).

Upon observing the ability of CD28 to manipulate Ras activation, we next investigated the hypothesis that activation of the Ras signaling pathway might be able to mimic some aspects of CD28-mediated TCR costimulation. In order to activate the Ras pathway in quiescent CD4^+^ effector cells, we transduced CAR transgenic (Tg) Th1 cells with an adenoviral vector encoding a constitutively active form of Ras (H-Ras61L). As shown in Figure 2A, activation of the Ras signaling pathway alone in these cells mimicked several biochemical consequences of CD28-costimulation by inducing AKT, JNK, and ERK phosphorylation.

Ligation of the TCR complex alone even with high concentrations of anti-CD3 mAb was not sufficient to induce significant IL-2 production by CAR Tg Th1 cells. However, CD28-mediated costimulation greatly augmented IL-2 secretion (Figure 2B). Interestingly, when these cells were transduced to express Ras61L, similar levels of IL-2 production were achieved in the presence of CD3 ligation alone, without CD28 co-ligation. Despite the partial overlap in signals emanating from CD28 and the TCR, no appreciable IL-2 production was noted in Ras61L-transduced cells stimulated only through CD28 (data not shown). The ability of active Ras to mimic CD28-mediated costimulation of IL-2 production was observed not only in CAR Tg Th1 T cell clones, but also in primary splenic CAR Tg CD4^+^ T cells (Figure 2C). This Ras-mediated costimulation was not a simple artifact of Ras overexpression, as transduction of these cells to express wild type H-Ras did not bypass the need for CD28 costimulation (Figure 2D). Additionally, H-Ras61L was relatively unique in its ability to replace CD28 costimulation as other deregulated mutant molecules that augmented IL-2 production in response to TCR/CD28 stimulation, such as dominant negative Cbl, were unable to replace the need for CD28 costimulation [22].

**Figure 3:**
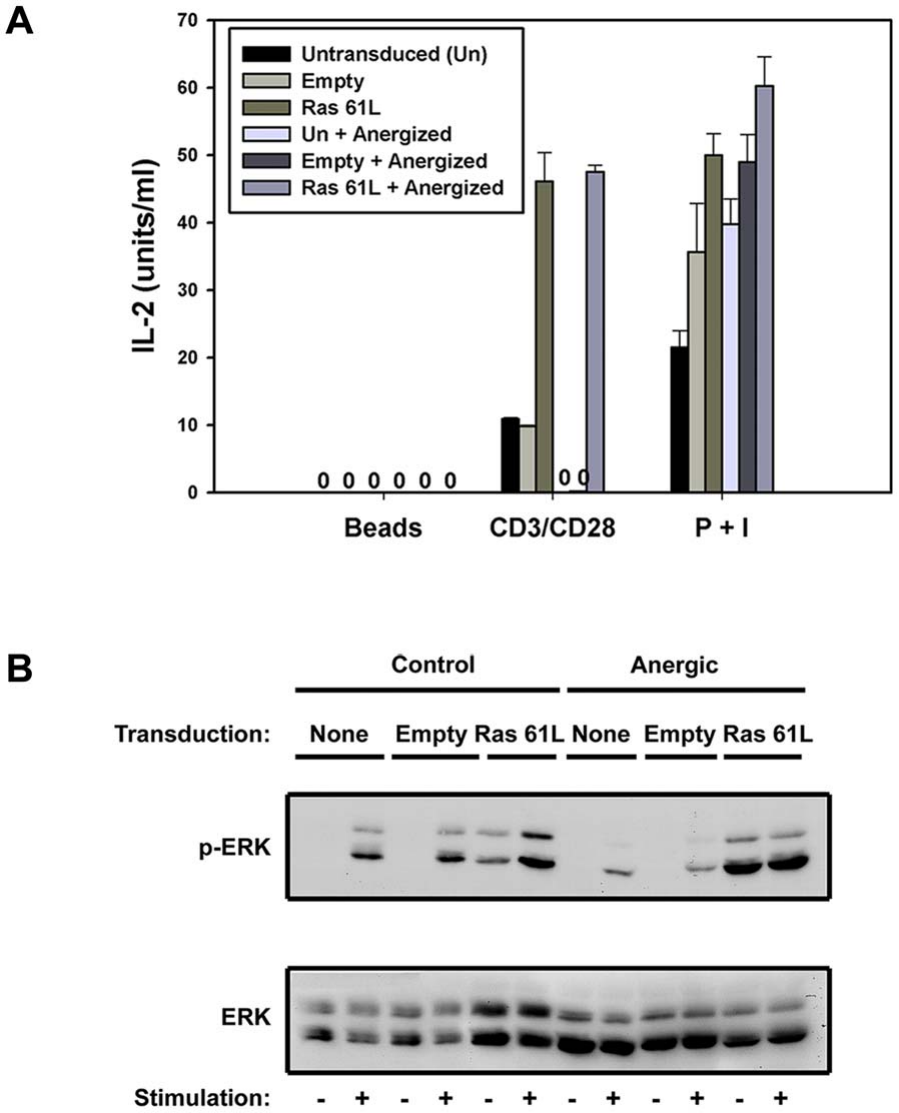
Active Ras can recapitulate CD28-mediated anergy prevention. **A.** CAR Tg Th1 cells were left untransduced or transduced with empty vector or Ras61L. They were then rested or submitted to anergy-inducing conditions as described in the materials and methods. **A.** These cells were then restimulated overnight with non-coated or antibody coated beads or PMA and ionomycin and supernatants were assessed for IL-2 production by ELISA. Error bars represent the standard deviation of mean values from triplicate samples from the same experiment. Cells transduced with active Ras prior to anergy induction produced significantly more IL-2 in response to CD3 and CD28 stimulation when compared to cells transduced with empty vector prior to anergy induction (p<0.05). **B.** Additionally, these cells were analyzed for pERK generation by western blot after 30-minute stimulation with anti-CD3 and anti-CD28 coated beads.

Besides potentiation of IL-2 production, CD28 costimulation has also been reported to prevent induction of T cell anergy, and to augment cell survival and glucose-based metabolism. Interestingly, the anergic state is itself associated with defective activation of Ras and its downstream effectors [23]. We have previously published that introduction of active Ras could restore IL-2 production in already anergized T cells [24]. By transducing CAR Tg T cells prior to anergy induction, we examined whether Ras activation could also *prevent* induction of anergy. As shown in Figure 3, introduction of active Ras indeed prevented anergy induction as reflected by preserved IL-2 production (A) and ERK phosphorylation (B) upon restimulation.

**Figure 4:**
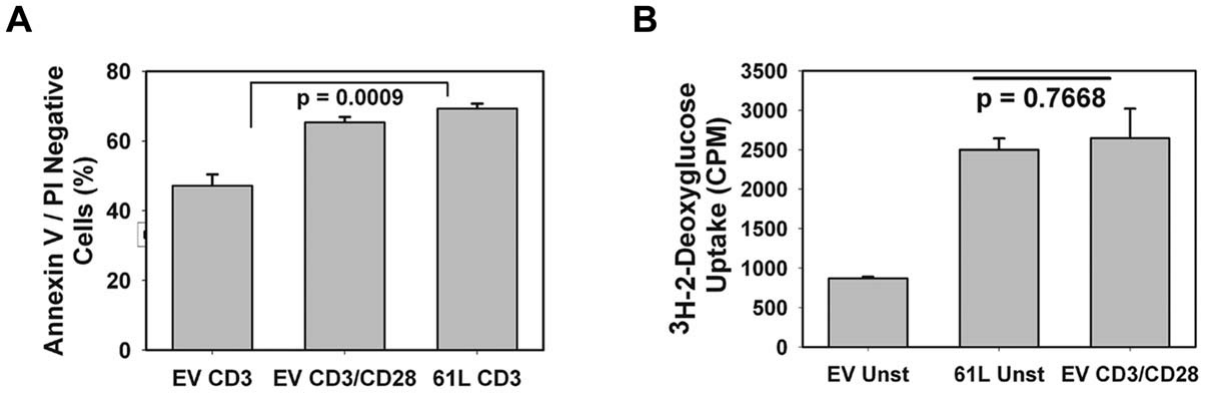
Active Ras can recapitulate CD28-mediated cell survival and increased glucose uptake. **A.** CAR Tg Th1 cells were transduced with empty vector or Ras61L and stimulated with antibody-coated beads in triplicate as indicated. Cells were analyzed for survival after 48 hours by staining with FITC-annexinV and propidium iodide followed by flow cytometry. P value indicates significantly increased AnnexinV/PI negative (live) cells when active Ras is added to CD3 stimulation. **B.** CAR Tg Th1 cells were transduced as in A and left unstimulated or stimulated in triplicate overnight (20 h) as indicated. Cells were pre-incubated in glucose uptake buffer and then exposed to ^3^H-2-deoxy-glucose (^3^H-2-DOG) for 2 minutes. ^3^H-2-DOG uptake was measured in a ß-scintillation counter. P value indicates no significant difference in glucose uptake by unstimulated Ras-transduced cells and empty vector-transduced cells stimulated with anti-CD3 and anti-CD28 antibodies. For all data shown in this figure, error bars represent standard deviation between triplicates samples from the same experiment.

We next examined the ability of CD28 or active Ras to augment cell survival. We confirmed that CD28-mediated co-ligation significantly augmented the survival of CD4^+^ effector cells stimulated through the TCR for 48 hours (Figure 4A). Interestingly, transduction with constitutively active Ras similarly augmented survival of TCR-stimulated T cells, indicating that Ras signaling could mimic this functional effect of CD28 as well.

Finally, we examined the ability of active Ras to mimic the ability of CD28 to costimulate cellular metabolism, as reflected by glucose uptake in concert with activation through the TCR. We transduced Ras61L into CARTh1 cells and measured uptake of tritiated-2-deoxy-glucose. As shown in Figure 4B, constitutive Ras signaling alone was able to increase glucose uptake to levels similar to those seen with stimulation through TCR and CD28. Importantly, CD3 stimulation of empty vector transduced cells induced maximal glucose uptake in our Th1 T cell clones without the need for CD28 costimulation (data not shown). This is likely due to the supraphysiologic concentrations of anti-CD3 and prolonged stimulation conditions required to measure glucose uptake assessment in this assay. Nonetheless, it is important that active Ras alone without any stimulation was able to induce the maximal amount of glucose uptake induced in stimulated cells, thus functionally mimicking any stimulation-induced changes in glucose uptake. Thus, expression of active Ras mimicked the ability of CD28 to augment IL-2 production, prevent anergy induction, potentiate T cell survival, and augment cellular metabolism.

### Transduction with Ras 61L increases both IL-2 promoter activity and IL-2 mRNA stability

**Figure 5:**
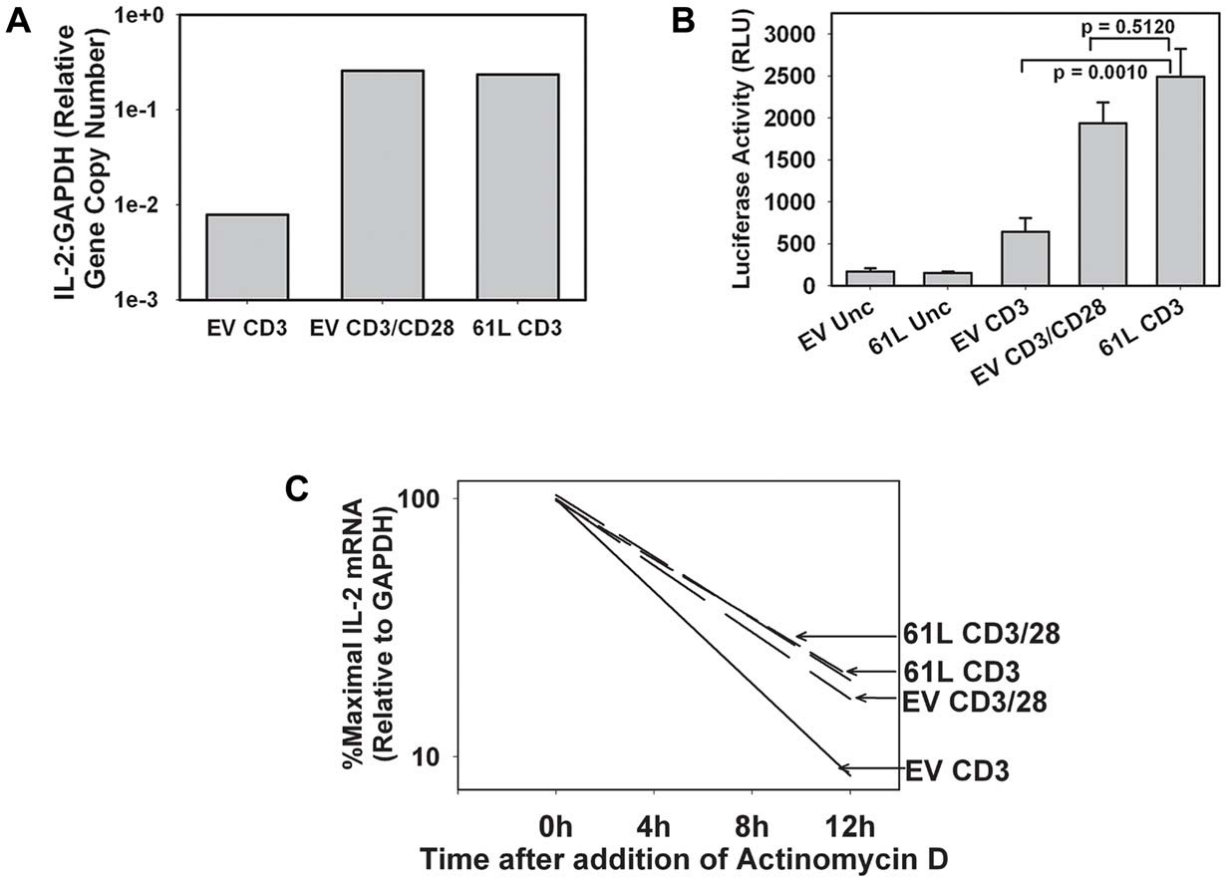
The mechanism of active Ras-mediated IL-2 costimulation mimics the mechanism of CD28-mediated costimulation. **A.** CAR Tg Th1 T cells were transduced and stimulated for four hours with antibody-coated beads as indicated. IL-2 mRNA production was assessed by real-time RT-PCR analysis of trizol lysates. **B.** CARTg/Luc Tg Th1 T cells expressing an IL-2 promoted-driven luciferase transgene were transduced and stimulated in triplicate overnight with antibody-coated beads as indicated. Luciferase activity was measured as a marker for IL-2 transcription as described in Materials and Methods. P values shown indicate a significant increase in luciferase activity when active Ras is added to CD3 stimulation and no significant difference in luciferase activity between Ras-transduced cells stimulated through CD3 alone and empty vector transduced cells stimulated through CD3 and CD28. **C.** CAR Tg Th1 T cells were transduced and stimulated as in A. Actinomycin D was added after 4 hours to stop further transcription. IL-2 mRNA levels were assessed at the time points indicated by real-time RT-PCR. Degradation curves were generated by linear regression modeling of real-time PCR data. Error bars shown indicate standard deviation of the mean between triplicate samples from the same experiment.

CD28 costimulates TCR-induced IL-2 production by increasing steady state IL-2 mRNA levels. This occurs both via increased transcription of the IL-2 gene as well as through increased stability of the transcribed IL-2 messenger RNA. In order to determine if the mechanism of Ras61L-mediated costimulation mimics the mechanism of CD28-mediated costimulation, we first assessed the steady state IL-2 mRNA levels of effector CD4^+^ T cells that were transduced with Ras61L or empty vector and stimulated through the TCR in the presence or absence of CD28 costimulation. Consistent with our ELISA data, both CD28 ligation and active Ras signaling augmented TCR-induced steady state IL-2 mRNA levels (Figure 5A). To assess effects on IL-2 promoter activity, similar experiments were done by transduction of Th1 cells derived from CAR Tg mice that had been crossed with IL-2 promoter-driven luciferase reporter mice, a system that allows analysis of IL-2 promoter activity in normal T cells. Both CD28-crosslinking and transduction with active Ras significantly and comparably augmented TCR-induced IL-2 promoter activity (Figure 5B). To determine the effects on mRNA stability, CAR Tg Th1 cells were similarly transduced and stimulated, and Actinomycin D was added after 4 hours to block further transcription. Real-time RT-PCR was then performed over time to determine the kinetics of IL-2 mRNA decay. As shown in Figure 5C, either ligation of CD28 or transduction with active Ras resulted in a similar prolongation of the IL-2 mRNA half-life. Therefore, the costimulation of IL-2 production mediated by active Ras occurs by a mechanism that mimics the mechanism of CD28-mediated costimulation, increasing both IL-2 transcription and mRNA stability.

### MEK signaling downstream of active Ras may be critical for its ability to costimulate IL-2 production

**Figure 6:**
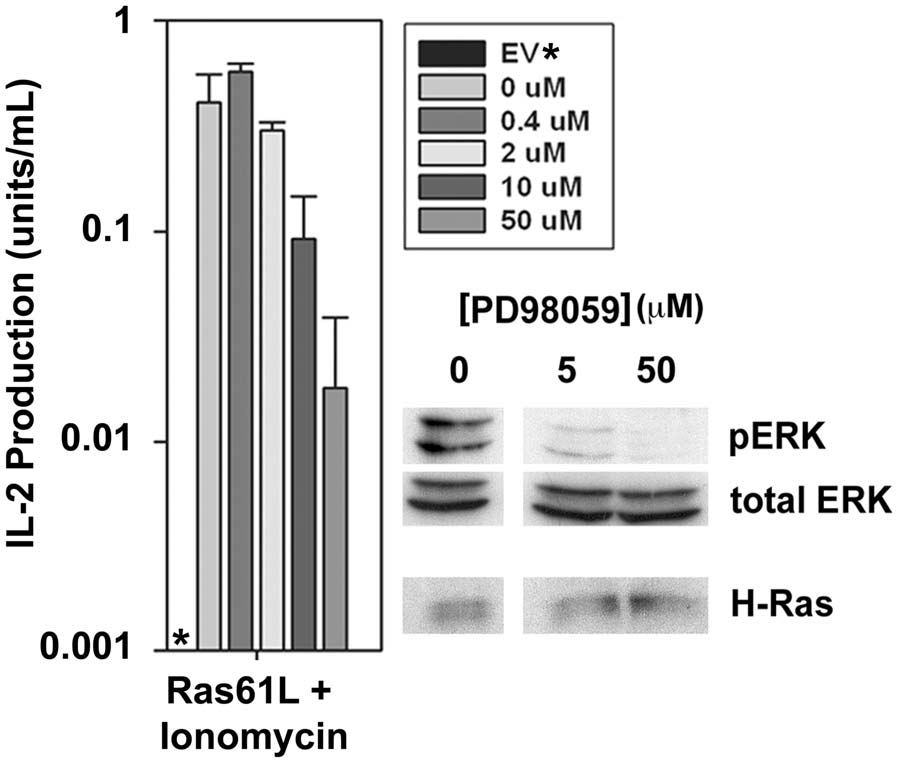
Ionomycin induced IL-2 production in Ra61L transduced cells is dependent on MEK signaling. CARTg Th1 T cells were transduced with active Ras and preincubated for 30 minutes with varying amounts of the MEK-1 inhibitor PD98059. Cells were then either stimulated with ionomycin (50 mg/mL) for 30 minutes and analyzed for ERK phosphorylation by Western blot, or stimulated with ionomycin overnight in triplicate and supernatants were collected and analyzed for IL-2 production by ELISA. Star (*) indicates no detectable IL-2 produced by empty vector transduced cells stimulated with ionomycin. Error bars where shown indicate standard deviations of the mean between triplicate samples from the same experiment.

Like CD28, active Ras engages multiple downstream pathways many of which are also activated by T cell receptor signaling. It therefore becomes difficult to dissect which pathway(s) downstream of active Ras may be contributing to its ability to costimulate IL-2 production and thus mimic CD28. One aspect of TCR signaling that cannot be reproduced by active Ras or CD28 but is critical to IL-2 production is induced elevation of intracellular free calcium. Similar to previous reports, we found that active Ras signaling induced IL-2 production independent of TCR stimulation when ionomycin was also provided to induce a calcium flux [25]. While this might not fully recapitulate the mechanisms of IL-2 production induced by TCR stimulation in conjunction with CD28 costimulation, it does allow determination of which signaling pathways downstream of active Ras may be critical for its ability to influence IL-2 production. We found that inhibition of the MEK/ERK MAP kinase signaling pathway using a pharmacologic inhibitor of MEK-1 caused a dose dependent inhibition in ERK phosphorylation and IL-2 production in Ras61L-transduced cells stimulated with ionomycin, arguing that this pathway is critical to the ability of Ras61L to costimulate IL-2 production (Figure 6).

## Discussion

The biochemical mechanism by which CD28 costimulation mediates the numerous described functional effects on T cells has remained enigmatic. The reasons for this are complex, and include the fact that CD28 readouts require concerted TCR engagement, that partial overlap exists between TCR, CD28 and Ras signaling pathways, and that several distinct model systems have been used ranging from T cell tumor lines to retrovirally-transduced murine activated T cells. However, recent evidence has resurrected the potential role for Ras being involved in this process. The results presented here demonstrate that active Ras can mimic CD28 for the key costimulatory functions of increasing IL-2 transcription and mRNA stability, preventing anergy, promoting cell survival, and augmenting glucose uptake. These results, combined with the observation that Ras activation is quantitatively and qualitatively modified by CD28 ligation, support a central role for Ras in CD28 costimulation.

Although the activation of Ras and its effectors AKT, ERK and JNK can be augmented by CD28, strong (likely supra-physiologic) TCR stimulation can also induce these biochemical changes without conferring the phenotypic changes imparted by CD28 costimulation. Our data argue that one explanation for this apparent paradox may be that CD28 may alter the intracellular compartment in which Ras is activated. Ras activation has been shown to occur in distinct compartments including the plasma membrane, golgi, endoplasmic reticulum [26], and even internalized vesicles [27], with functionally distinct effects (reviewed in [28], [29]). Therefore, by promoting the location of Ras activation to the T cell-APC interface, it is possible that CD28 costimulation may influence the functional outcome of the Ras pool that is activated. Although this correlation between CD28 costimulation and RasGRP recruitment to the T cell/APC interface is compelling, a causal relationship has not yet been established. One potential area of future study will be to investigate whether forcing RasGRP localization to the plasma membrane can also impart a partial CD28-independence, and conversely whether prevention of RasGRP membrane localization would attenuate the effects of CD28 costimulation.

Interestingly, we noted that RasGRP1 expressed in the P815.B7 antigen presenting cells was also occasionally recruited to the T cell-APC interface. However, this was not regularly reproducible as it was in the 2C T cells. The nature of RasGRP1 localization and mobilization in the P815.B7 cell may be an interesting area of future study and could potentially occur through reverse B7 signaling in this mastocytoma-derived cell line, as has been demonstrated previously in dendritic cells [30], [31], [32].

Several hypothetical mechanisms exist to explain why changing the subcellular localization of Ras activation may alter the functional output from the pathway. First, it may place Ras activation in the context of a distinct set of downstream signaling effectors. The notion of CD28 costimulation modifying the effectors engaged by Ras is supported by previous published work. Cofilin dephosphorylation is a critical step in T cell activation and immune synapse formation [33], and is mediated by Ras signaling [34]. However, a series of studies have suggested that this dephosphorylation occurs in response to TCR plus CD28 costimulation, but not in response to TCR stimulation alone, indicating that Ras-mediated cofilin dephosphorylation only occurs in the context of CD28 costimulation [35], [36], [37], [38]. Second, the isoform(s) of Ras activated with CD28 costimulation could theoretically be partially distinct. T cells contain at least three isoforms of Ras, H-Ras, K-Ras, and N-Ras. Activation of each of these isoforms can result in partially distinct effector programs (reviewed in [39]). However, our preliminary data suggests that the same degree of activation of each Ras isoform occurs whether or not CD28 is co-ligated with the TCR complex (data not shown). Another possibility is that CD28 could alter the dominant guanine nucleotide exchange factor mediating Ras activation or the intracellular localization of said GEFs. Our data demonstrate relocalization RasGRP to the plasma membrane, potentially due to DAG enrichment at the TCR-APC interface. In addition, as stated in the introduction, the ability of CD28 to recruit Grb2/SOS complexes through direct binding to its cytoplasmic tail may also contribute to costimulatory function. Considerable investigation will be necessary to investigate these multiple possibilities which are intriguing areas future study.

The ability to mimic the biochemical consequences of CD28 costimulation has potential therapeutic implications. CD28 costimulation has been shown to augment immune responses against tumors [40], [41], viruses (reviewed in [42]), and other pathogens in vivo. Conversely, lack of costimulation in vivo has been shown to be a critical component of failed immune responses against tumors [43], and also in promoting tolerance in models of transplantation and autoimmunity [44], [45]. Understanding the detailed biochemical mechanisms by which CD28 and Ras control productive T cell activation versus anergy in vivo may enable the development of pharmacologic compounds that either augment or inhibit Ras-based signaling in T cells. Such agents could have utility in either potentiating T cell responses against tumors and pathogens, or suppressing T cell responses in autoimmunity or transplantation.

## Materials and Methods

### Ethics statement

This study was carried out in strict accordance with the recommendations in the Guide for the Care and Use of Laboratory Animals of the National Institutes of Health. All protocols used for animal breeding and experimentation in this study were approved by the Institutional Animal Care and Use Committee of the University of Chicago (Protocol numbers 71585, 71586, 71954).

### Mice and cell lines

CAR transgenic (Tg) mice were generated as described [19] and were interbred with IL-2-promoter/luciferase reporter (IL-2/Luc) transgenic mice [46] (kindly provided by Dr. Jim Miller, University of Rochester) to generate CAR Tg × IL-2/Luc double Tg mice. OVA-reactive CAR Tg and CAR Tg × IL-2/Luc Th1 clones were generated and maintained as described [47]. Mice were maintained in specific pathogen–free conditions in a barrier facility at the University of Chicago (Chicago, IL).

### Antibodies and adenoviral vectors

The antibody against H-Ras (259) was purchased from Santa Cruz Technologies (Santa Cruz, CA). Anti-pAKT (9271) and anti-pERK (9101) were purchased from Cell Signaling (Danvers, MA). Anti-total ERK (13-6200) was purchased from Zymed Technologies (Invitrogen) (Carlsbad, CA). Anti-pJNK(V793A) antibody was purchased from Promega, and anti–JNK1 (554286) was purchased from BD Pharmingen (San Jose, CA). The anti-CD3 and anti-CD28 mAbs were purified from the 145-2C11 and PV-1 hybridomas, respectively. The B7-1-Ig fusion protein was a gift from Genetics Institute (Cambridge, MA). Adenoviral vectors expressing either no insert, H-Ras61L, or WT H-Ras driven by the human Ubiquitin C promoter were generated as described [19], [48].

### Adenoviral transduction

CARTg Th1 cells were incubated at high cell density (10^7^/mL) with recombinant adenoviruses in 2% FCS DMEM in Eppendorf tubes for 1 hour followed by overnight resting in 5% FCS at low cell density (4×10^5^/mL). For experiments using splenic T cells, cells were rested for 4 days in IL-7 (1 ng/mL) to allow sufficient time for gene expression. Following overnight rest, the cells were washed to remove any residual virus for use in experiments.

### Cell stimulation and Western blotting

Antibody-coated stimulation beads were prepared by incubating sheep anti-mouse M450 Dynabeads (Dynal Biotech (Invitrogen) Carlsbad, CA) (50×10^6^/mL) with anti-CD3 (0.01–1 µg/mL) and/or anti-CD28 (2 µg/mL) or B7-1Ig fusion (as indicated) in 0.5% BSA containing Ca^++^/Mg^++^ free PBS for 2 hours at room temperature, followed by washing. Cells were stimulated at a ratio of 5 beads per 1 T cell or with PMA (50 mg/mL) and/or ionomycin (500 mg/mL) mixed in pre-warmed culture cell culture media. For inhibitor studies, the cells were treated with the MEK-1 inhibitor, PD98059, (BD Pharmingen) in warmed complete media prior to (30 minute pre-incubation) and during stimulation for ELISA and biochemical analysis.

For biochemical analysis, cells were stimulated at 10×10^6^cells/mL for 30 minutes in pre-warmed complete medium. After quenching and washing in ice cold Ca^++^/Mg^++^-free PBS, cells were lysed in 0.5% Triton X-100 lysis buffer and analyzed by Western blot using the appropriate antibodies.

### Ras activation assay

20×10^6^ CARTg Th1 T cells were stimulated as described for biochemical analysis. Cells were then lysed and analyzed for Ras activation using the EZ-Detect Ras Activation Kit (Pierce (Rockford, IL)) according to the manufacturer's protocol.

### Cytokine ELISAs

10^5^ CAR Tg Th1 T cells or splenic CD4^+^ T cells were transduced and seeded in triplicate in 96-well plates and stimulated with antibody-coated beads overnight. Supernatants were harvested and analyzed for IL-2 by ELISA using antibody pairs from BD Pharmingen (San Jose, CA).

### Induction of T cell anergy

Transduced Th1 cells were anergized in vitro by stimulation with plate-bound anti-CD3 Ab for 24–48 hours, harvested, and rested in culture medium alone for 24–72 hours as described [23]. Defective IL-2 production in response to CD3/CD28 ligation but not with PMA + Ionomycin was used to confirm an anergic state.

### Assessment of T cell survival

10^6^ CARTg Th1 T cells were transduced, seeded in 24-well plates, and stimulated with antibody-coated beads for 48 hours. Cells were harvested and stained with FITC-annexinV and propidium iodide (BD Pharmingen (San Jose, CA)) according to the manufacturer's protocol and analyzed by flow cytometry. Surviving cells were defined as AnnexinV/PI-negative.

### mRNA analysis and luciferase assays

For steady state IL-2 mRNA assessment, 2.5×10^6^ CARTg Th1 T cells were seeded in 6-well plates and stimulated with antibody-coated beads for 4 hours. Cells were lysed in Trizol reagent (Gibco (Invitrogen, Carlsbad, CA)), RNA was isolated, and cDNA was synthesized using MMLV-RT (Invitrogen, Carlsbad, CA). Real-time PCR was then performed using IL-2 and GAPDH primer and probe sets (Applied Biosystems, Foster City, CA) according to manufacturers' protocols. For IL-2 mRNA stability analysis, cells were stimulated as above for 4 hours, followed by the addition of Actinomycin D (5 µg/mL) to prevent further transcription. Cells were lysed and analyzed for IL-2 mRNA at 4, 8, and 12 hours by real time RT-PCR. mRNA degradation curves were generated by linear regression modeling using Sigmal Plot analysis software (Systat Software, San Jose, CA). IL-2 promoter activity was assessed using 10^6^ CARLucTh1 T cells stimulated overnight in a 96-well plate with antibody-coated beads under identical conditions to those used for ELISA. Luciferase activity was measured using Bright-Glo Luciferase Assay System (Promega, Madison, WI).

### Glucose uptake assays

CARTg Th1 clones were were seeded in 6 well plates at 2.5×10^6^ cells/well and stimulated for 20 h with antibody coated beads. Glucose uptake was measured as described [3], [49].

### Immunofluorescent confocal microscopy

Analysis of protein localization in 2C T cell/P815 cell conjugates was performed as described previously [15], [50]. Briefly, P815 cells stably transfected with control vector or B7.1 were labeled with 7-amino-4-chloromethylcoumarin (CMAC) Cell-Tracker Blue (Molecular Probes) and were mixed at equal numbers with in vitro primed 2C/RAG2^−/−^ T cells. After approximately 8 total minutes, cells were fixed, permeabilized, and stained with anti-RasGRP1and anti-talin (both from Santa Cruz), with species-specific secondary antibodies conjugated to FITC or PE, respectively. Samples were analyzed by using a Zeiss Axiovert 100 microscope, and 100 conjugates were scored for statistical analysis. Image capture and deconvolution analysis were performed by 24 using Slidebook software (Intelligent Imaging Innovations, Denver). T cell-APC conjugates were defined as CMAC-labeled APCs in contact with a T cell displaying talin localized to the T cell–APC interface. The T cell-APC interface was defined as the area of talin localization seen on immunofluorescent staining that corresponded to an area of T cell-APC contact seen on bright field imaging. Because of the irregular and unique shape of each interface, conjugate specific polygons were drawn to measure and define the APC-interface used for further image analysis using Image J 1.236b software (National Institute of Health). The T cell cytoplasm area was defined as the area not corresponding to the T-cell APC interface that still fell within the area occupied by the T cell on bright field imaging. As above, conjugate-specific polygons were drawn for each T cell to define the area of cytoplasm used for further image analysis. The ratio of RasGRP localizing to the T cell-APC interface versus the cytoplasm was calculated by measuring the pixel intensity of RasGRP staining at the T cell interface versus the T cell cytoplasm. Quantification of pixel intensities was done using ImageJ 1.36b software.

### Statistical Data Analysis

All p-values noted in this study were calculated using an unpaired student's t-test. The values being compared are noted in the figure legends or text. Error bars in the figures represent standard deviations of the mean value being represented in the figure (the specific mean values being analyzed are noted in the figure legends or text). All experiments presented within this study are reflective of at least 3 replicate studies with similar results.
